# Iron-Storage Protein Ferritin Is Upregulated in Endometriosis and Iron Overload Contributes to a Migratory Phenotype

**DOI:** 10.3390/biomedicines8110454

**Published:** 2020-10-27

**Authors:** Jeong-Hwa Woo, Youn Seok Choi, Jung-Hye Choi

**Affiliations:** 1 College of Pharmacy, Kyung Hee University, Seoul 02447, Korea; jhwoo@khu.ac.kr; 2 Department of Obstetrics and Gynecology, School of Medicine, Catholic University of Daegu, Daegu 42472, Korea; drcys@cu.ac.kr; 3 Department of Life and Nanopharmaceutical Sciences, Kyung Hee University, Seoul 02447, Korea

**Keywords:** Endometriosis, iron, migration, ROS, NFκB

## Abstract

High levels of iron in the peritoneal cavity during menstruation have been implicated in the pathogenesis of endometriosis. However, whether iron directly affects the growth or migration of human endometriotic cells is poorly understood. This study demonstrated the presence of increased levels of the iron storage protein, ferritin, in the endometriotic tissues of patients with endometriosis. Furthermore, iron treatment stimulated the migration and epithelial–mesenchymal transition (EMT), but not growth, of 12Z human endometriotic cells. The expression of matrix metalloproteinase (MMP)-2/-9 was markedly increased through iron treatment in 12Z cells. Interestingly, intracellular reactive oxygen species (ROS) levels were significantly increased by iron in 12Z cells, and N-acetyl-L-cysteine significantly reduced iron-induced migration and MMP-2/-9 expression. Additionally, iron stimulated the activation of the NFκB pathway, and the activation was associated with iron-induced migration and MMP-2/-9 expression in 12Z cells. Moreover, iron markedly increased EMT and MMP-2/-9 expression in endometriotic lesions in an endometriosis mouse model. Taken together, these results suggest that iron may contribute to the migration abilities of human endometriotic cells via MMP expression through the ROS–NFκB pathway.

## 1. Introduction

Endometriosis is a chronic disease of adolescents and reproductive-aged women, characterized by the presence and growth of endometrial tissue outside the uterus [1]. Despite many studies, the physiopathology of endometriosis remains elusive due to its multifactorial characteristics [2]. Steroid hormones have been implicated in endometriosis [3]. In particular, the functional dysregulation of estrogen and progesterone play a critical role in the pathogenesis of endometriosis, through excessive estrogenic action and progesterone resistance [4]. In fact, the most widely utilized treatments for endometriosis are applied to suppress either estrogen production or its action. Recent studies have suggested that iron overload may play a role in the pathophysiology of endometriosis [5]. Iron is an essential factor for body homeostasis and participates in the regulation of cell survival [6]. However, excess free iron catalyzes reactions that result in the formation of ROS and oxidative stress [7]. Oxidative stress caused by iron overload has been implicated in the pathogenesis of numerous human diseases, including cancer [8], cardiac disease [9], diabetes [10], and neurodegenerative diseases [11,12]. Furthermore, iron overload could affect a wide range of mechanisms involved in endometriosis development [5]. According to Sampson’s implantation theory, retrograde menstruation appears to be greater in patients with endometriosis than in women without endometriosis [13]. This may result in the increased reflux of erythrocytes into the pelvic cavity in such patients, leading to iron overload in the different compartments (lesions, peritoneal fluid, and peritoneal macrophages) [7]. In fact, previous studies have reported that increased levels of iron and the iron storage protein, ferritin, were observed in the peritoneal fluid of patients with endometriosis [14,15]. Although these data suggest a potential role of iron overload in endometriosis pathogenesis, the specific function of iron in endometriosis is poorly characterized.

Endometriosis is not a cancerous disease, but endometriotic lesions have cancer-like traits, including cell invasion and migration [16]. Endometriotic epithelial cell lines, such as 12Z, have shown enhanced migratory potential compared to normal endometrial epithelial cells [17]. The elevated expression of epithelial–mesenchymal transition (EMT) inducer Snail and Slug has been observed in eutopic endometrium [18], and estrogen has been demonstrated to induce EMT in endometriosis [18,19,20]. These findings suggest a role of EMT in the development and pathogenesis of endometriosis. Additionally, matrix metalloproteinases (MMPs) have been suggested to play a role in endometriosis owing to their characteristics of enhancing the migration and invasion abilities of endometrial cells [21,22,23,24,25,26]. Here, we investigated the effect of iron on cell migration and MMP-2/-9 and EMT marker expression in human endometriotic cells, and the underlying molecular mechanism of action.

## 2. Materials and Methods

### 2.1. Cells and Materials

The human endometriotic cells (12Z) were a generous gift from Dr Starzinski-Powitz (Johann-Wolfgang-Goethe-Universitaet, Germany) and were cultured in DMEM supplemented with 10% fetal bovine serum (FBS), streptomycin sulfate (100 μg/mL) and penicillin (100 U/mL). DMEM medium, FBS, streptomycin sulfate and penicillin were procured from Life Technologies, Inc. (Grand Island, NY, USA). FeSO_4_·7H_2_O, estradiol valerate, Bay 11-7082, N-acetyl-l-cysteine (NAC), and carboxymethylcellulose sodium salt (CMC) were obtained from Sigma Chemical (St. Louis, MO, USA). Ferric ammonium citrate (FAC) was obtained from Thermo Fisher Scientific Korea Ltd. (Seoul, Korea). 2-[[(4-Phenoxyphenyl)sulfonyl]methyl]thiirane (SB-3CT), a gelatinase (MMP-2 and MMP-9) inhibitor, was purchased from EMD Millipore (Temecula, CA, USA).

### 2.2. Bioinformatic Analysis

To evaluate the expression levels of ferritin in endometriotic tissues from patients with endometriosis, four datasets containing an expression profile of ferritin were selected from the Gene Expression Omnibus (GEO) database (http://www.ncbi.nlm.nih.gov/geo/). GSE25628 included 7 ectopic and 9 eutopic endometrium tissues from patients with endometriosis and 6 control endometrium tissues. GSE23339 included 10 endometrioma and 9 control endometrium tissues, while GSE7305 included 10 endometrium tissues from patients with endometriosis and 10 control endometrium tissues, and GSE7846 included 5 eutopic endometrium tissues from patients with endometriosis and 5 control endometrium tissues.

### 2.3. Transwell Migration Assay

Polyvinylpyrrolidone-free polycarbonate filters (8 µm pore size) were used for transwell migration assay. After the harvesting of cells with trypsin, cells were suspended in 1% FBS DMEM medium with different concentrations of iron (FeSO_4_: 50, 100 and 200 µM; FAC: 50, 100 and 200 μg/mL) in the presence or absence of pretreatment with inhibitors. The mixtures were placed in the upper chambers. The lower chambers were filled with DMEM medium containing 5% FBS. After 24 h, the inserts were removed and the inner side was wiped with cotton swaps. Following methanol fixation and staining with 0.5% (*w*/*v*) crystal violet (BD Biosciences, San Diego, CA, USA), migrated cells in the lower surface of the membrane were quantified under a microscope. Experiments were done in triplicate, and a minimum of five fields per filter was counted.

### 2.4. Measurement of Intracellular Reactive Oxygen Species

After treatment of endometriotic cells with iron, the cells were suspended in ice-cold PBS. After being washed twice with ice-cold PBS, the cells were suspended with 100 µL of ice-cold PBS and stained with DCF-DA (20 mM). Then, cells were incubated in the dark at room temperature for 20 min. The cells were centrifuged and ROS production was measured with a Guava easy Cyte flow cytometry system (guava easy cyte™, Merk Millipore, Germany).

### 2.5. Western Blot Analysis

For whole cell lysates, cells were rinsed twice with cold PBS and mixed with a protein lysis buffer (Intron Biotechnology, Seoul, Korea) containing protease inhibitors (0.5 mM phenylmethylsulfonylfluoride and 5 µg/mL aprotinin). Phenylmethylsulfonylfluoride and aprotinin were procured from BD Biosciences (San Diego, CA, USA). For nuclear fractions, cells were suspended in 100 µL of hypotonic buffer A (10 mM HEPES (pH 7.8), 1.5 mM MgCl_2_, 10 mM KCl, 0.5 mM DTT, and 0.2 mM PMSF) for 15 min on ice, and 1 µL of 10% Nonidet P-40 solution was added. The mixture was centrifuged at 13,000 rpm for 10 min at 4 °C. The pellets were washed with hypotonic buffer and resuspended in hypertonic buffer C (20 mM HEPES (pH 7.8)), 20% glycerol, 420 mM NaCl, 1.5 mM MgCl_2_, 0.2 mM EDTA, 0.5 mM DTT, 0.2 mM PMSF) for 40 min on ice and centrifuged at 13,000 rpm for 10 min at 4 °C. The supernatant containing nuclear proteins was collected. PARP (poly(ADP-ribose) polymerase) was used as a nuclear faction marker. After protein quantification using Bradford assay, the lysate was denatured with the SDS-PAGE sample buffer followed by 5 min boiling at 95 °C. Total protein (30 µg) was used for SDS-PAGE and the separated proteins were blotted onto polyvinylidene difluoride (PVDF) membrane from the gel. The membrane was post-coated with 5% skimmed milk in Tris-buffered saline (Boster Biological Technology Ltd., Wuhan, China) containing Tween-20 for 1 h. After incubation overnight at 4 °C with the diluted corresponding primary antibodies, the membrane was incubated with optimal dilution of the appropriate horseradish peroxidase-linked secondary antibody for 2 h at room temperature. Ferritin, MMP-9, PARP, p65, vimentin, and β-actin antibodies were procured from Santa Cruz Biotechnology. MMP-2, p-p65 and E-cadherin antibodies were obtained from Cell Signaling Technology (Danvers, MA, USA). Secondary antibodies were obtained from The Jackson Laboratory (West Grove, PA, USA). After incubation with enhanced chemiluminescence (ECL) solution (EMD Millipore, Billerica, MA, USA), the signals for the immunoreactive bands were analyzed by Image Quant LAS-4000 (Fujifilm Life Science, Tokyo, Japan).

### 2.6. Real-Time RT-PCR Analysis

Reverse transcription of total RNA (1 µg) was performed using a First-Strand cDNA synthesis kit based on the manufacturer’s instruction. Total RNA was extracted using Easy Blue kit. The cDNA was amplified using Thermal Cycler Dice Real Time PCR system and SYBR Premix Ex Taq (Takara, Tokyo, Japan). A dissociation curve analysis revealed a single peak. The expression of the gene of interest was analyzed using the comparative Ct method, in which the mean Ct of the target cDNA is normalized to that of a reference gene, GAPDH. The sequences of the primers used for real-time RT-PCR were as follow: for MMP-2, sense primer, 5′-ACC GCG AGA AGT ATG GC-3′, and anti-sense primer, 5′-CCA CTT GCG GTC ATC AT GT-3′; for MMP-9, sense primer, 5′-CGA TGA CGA GTT GTG GTC CC-3′, and anti-sense primer, 5′-TCG TAG TTG GCC GTG GTA CT-3′.

### 2.7. Luciferase Activity Analysis

12Z cells were co-transfected with NFκB-luc reporter plasmid and phRL-TK plasmid (Promega, Madison, WI, USA) using Lipofectamine LTX™ (Invitrogen, Carlsbad, CA, USA) according to the manufacturer’s instruction. At 24 h after transfection, cells were treated with iron for 12 h. The cells were lysed and the luciferase activities were determined using the Promega luciferase assay system (Promega, Madison, WI, USA) according to the manufacturer’s instructions.

### 2.8. Animal Study

The female mice (5 weeks of age, BALB/c) used in the study were purchased from Korea Orient Bio, Inc. (Seoul, Korea). After the adaptation periods of 7 days, the mice were housed in separate cages at an ambient temperature of relative humidity 55 ± 5% and 24 ± 1 °C under a 12 h light/dark cycle and were allowed free access to water and food. All animal studies were performed in accordance with institutional guidelines for laboratory animal care and approved by the “Animal Care and Use Guidelines” of Kyung Hee University (the approval number: KHSASP-19-303). Endometriotic lesions was induced in the peritoneal cavity of mice according to a previously developed method with modifications [27,28]. Briefly, after removal of the uterine horns of the donor mice, the endometrium-rich fragment (1 cm) of the middle-third of the uterine horn was uniformly and finely chopped. To induce the formation of endometriosis-like lesions, the fragments (~20 pieces) suspended in PBS were intraperitoneally injected with a micropipette in recipient mice. To stimulate the growth of endometriotic lesion [3], estradiol valerate (2 µg/mouse) was given intraperitoneally every week from the time of endometriosis induction. After 3 days, the 9 mice were randomly divided into three groups (3 mice/group): vehicle (0.05% CMC), FAC (15 mg/kg), and FAC (75 mg/kg). The mice were intraperitoneally injected with vehicle or FAC three times per week for 5 weeks. After 6 weeks from induction, the mice were sacrificed by cervical dislocation and the visceral and peritoneum organs were evaluated visually to examine the number of endometriotic lesions (>1 mm). Mice body weight changes were measured once per week

### 2.9. Statistical Analysis

Statistical analysis was conducted using a one-way ANOVA analysis or Student *t*-test. A *p*-value of less than 0.05 was considered statistically significant.

## 3. Results

### 3.1. Expression of Ferritin in Endometriotic Tissues

**Figure 1:**
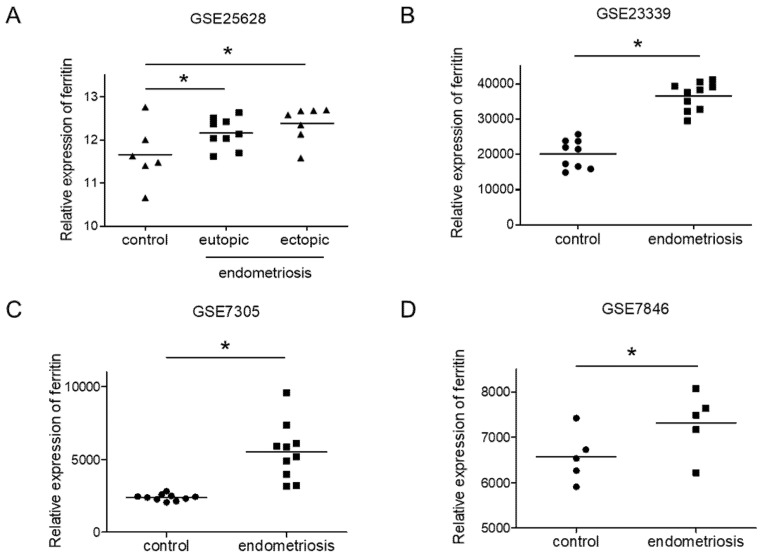
Expression of ferritin in endometriotic tissues. Ferritin expression was evaluated using four GEO datasets: GSE25628 (**A**), GSE23339 (**B**), GSE7305 (**C**) and GSE7846 (**D**). The values represent the mean ± SD of results. * *p* < 0.05.

To validate the potential role of iron in the development of endometriosis, we analyzed the expression of ferritin, an intracellular iron-storage protein that plays a key role in iron metabolism [29], in endometriotic tissues. We used four GEO datasets (GSE25628, GSE23339, GSE7305 and GSE7846) to compare the ferritin expression between patient endometriotic tissues and control endometrium tissues. In the GSE25638 dataset, the ferritin expression in ectopic endometriotic tissues was not different from that in eutopic tissues (Figure 1A). In contrast, in all four datasets, the ferritin levels were significantly enhanced in endometriotic tissues compared to control (Figure 1A–D). This finding is consistent with a previous study showing increased iron and ferritin levels in the peritoneal fluid of patients with endometriosis, and higher rates of iron deposits in the peritoneal tissue adjacent to lesions [15,30]. Together, these data suggest that iron may play a role in endometriosis.

### 3.2. Effect of Iron on Migration in Human Endometriotic Cells

**Figure 2:**
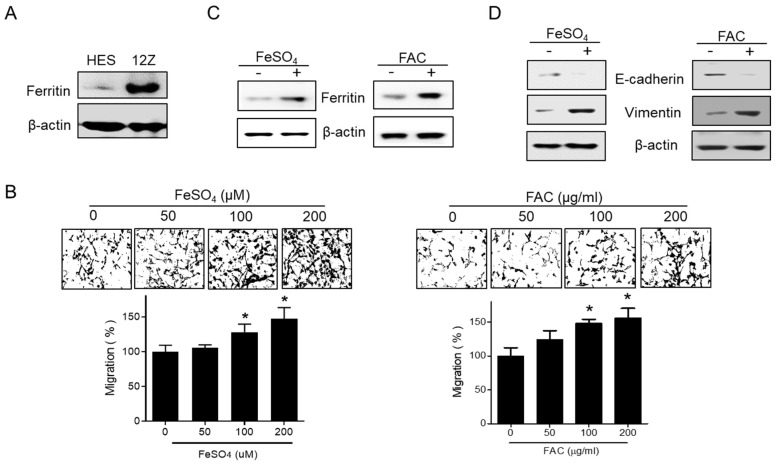
Effect of iron on migration in human endometriotic cells. (**A**) The protein expression of ferritin was investigated by Western blot analysis in human endometrial HES and endometriotic 12Z cells. (**B**) 12Z cells were treated with iron (FeSO_4_: 50, 100, and 200 µM; FAC: 50, 100, and 200 µg/mL). The cells were allowed to migrate for 24 h. Transwell migration assay was performed. Values represent the means ± SD of three independent experiments. * *p* < 0.05 as compared with control. (**C**,**D**) 12Z cells were treated with iron (FeSO_4_: 100 µM; FAC: 100 µg/mL) for 24 h. The protein expressions of ferritin, E-cadherin, and vimentin were determined by Western blot analysis. β-Actin was used as a loading control. Representative images of three independent experiments are shown.

The expression of ferritin was also assessed in human endometrial HES and human endometriotic 12Z cells. 12Z cells showed high levels of ferritin when compared with HES cells (Figure 2A). To investigate the specific function of iron in the pathogenesis of endometriosis, FeSO_4_ and ferric ammonium citrate (FAC) were used to induce iron overload conditions in 12Z cells and cell viability was examined. FeSO_4_ (50, 100, and 200 µM) and FAC (50, 100, and 200 µg/mL) had no significant effect on cell viability in 12Z cells (Supplementary Figure S1). Next, we investigated the effect of iron treatment on 12Z cell migration. Notably, both FeSO_4_ and FAC significantly increased the transwell migration of 12Z cells at a concentration that did not affect cell viability (Figure 2B). The levels of ferritin were enhanced in 12Z cells by the FeSO_4_ and FAC treatment (Figure 2C). Moreover, FeSO_4_ and FAC decreased the expression of E-cadherin, and increased the expression of vimentin, suggesting that FeSO_4_ and FAC treatment induces EMT, resulting in enhanced migration capability (Figure 2D). These data suggest that the elevated levels of iron found in the peritoneal fluid of women with endometriosis may promote the migration of endometriotic cells.

### 3.3. Effect of Iron on Matrix Metalloproteinase-2/-9 Expression in Human Endometriotic Cells

**Figure 3:**
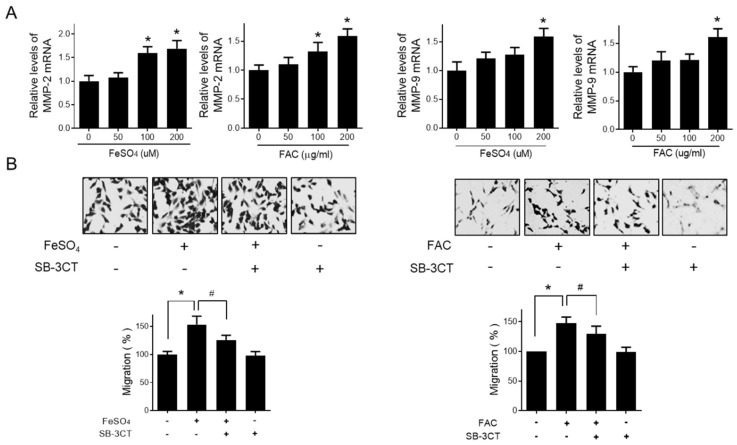
Effect of iron on MMP expression in human endometriotic cells. Human endometriotic 12Z cells were treated with iron (FeSO_4_: 50, 100, and 200 µM; FAC: 50, 100, and 200 µg/mL) for 24 h. (**A**) Real-time RT-PCR was performed to measure the mRNA levels of MMP-2 and MMP-9 in 12Z cells. (**B**) 12Z cells were treated with iron (FeSO_4_: 100 µM, FAC: 100 µg/mL) for 24 h in the presence of 10 µM of SB-3CT, a gelatinase (MMP-2 and MMP-9) inhibitor. Transwell migration assay was performed. Representative images of three independent experiments are shown. Values represent the means ± SDs of three independent experiments. * *p* < 0.05 as compared the control group; # *p* < 0.05 as compared the treated group.

Gelatinases (MMP-2 and MMP-9) are known to be the major proteinases that contribute to the metastatic ability of endometriotic cells to infiltrate the basement membrane [31,32]. Thus, we investigated the role of MMP-2 and MMP-9 in the iron-induced migration of endometriotic cells. As shown in Figure 3A, the mRNA levels of MMP-2 and MMP-9 were significantly increased by FeSO_4_ and FAC treatment. We confirmed the involvement of MMP-2 and MMP-9 in iron-stimulated migration using SB-3CT, a specific gelatinase inhibitor (Figure 3B). SB-3CT significantly suppressed the iron-stimulated migration of endometriotic 12Z cells. These results suggest that iron promotes the migration of endometriotic cells by regulating MMP-2/-9 expression.

### 3.4. Involvement of Reactive Oxygen Species (ROS) in Iron-Induced Migration

**Figure 4:**
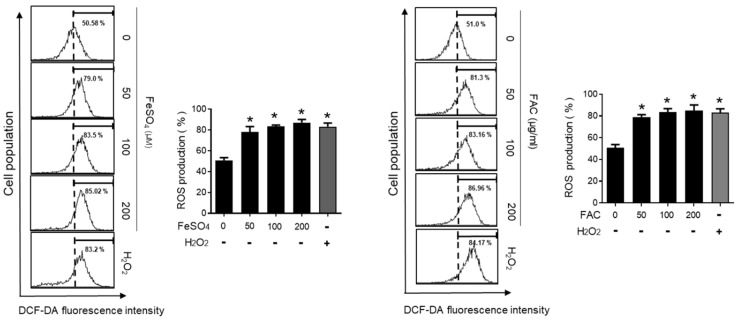
Effect of iron on the levels of intracellular ROS in human endometriotic cells. Human endometriotic 12Z cells were treated with iron (FeSO_4_: 50, 100, and 200 µM; FAC: 50, 100, and 200 µg/mL) for 24 h. Intracellular levels of ROS were determined by fluorescence-sensitive probe DCF-DA assays. H_2_O_2_ (50 µM) was used as a positive control. The values represent the mean ± SD of results from three independent experiments. * *p* < 0.05 as compared with control.

**Figure 5:**
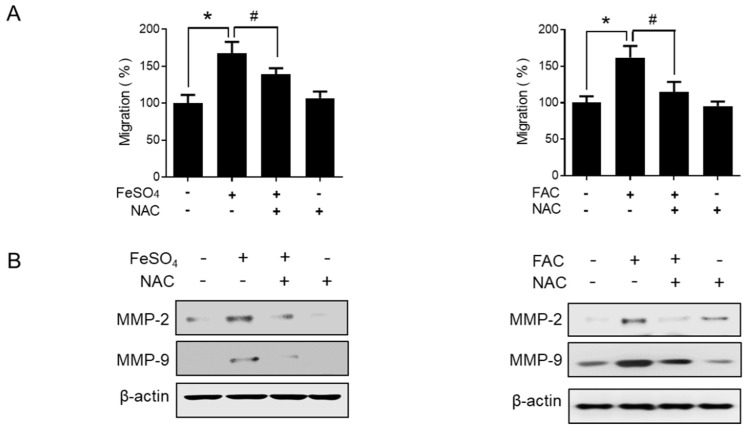
Effect of NAC on iron-induced migration in human endometriotic cells. Human endometriotic 12Z cells were treated with iron (FeSO_4_: 100 µM; FAC: 100 µg/mL) in the presence of 5 mM of N-acetyl-l-cysteine (NAC) for 24 h. (**A**) Transwell migration assay was performed. (**B**) The protein expressions of MMP-2 and MMP-9 were determined by Western blot analysis. β-Actin was used as a loading control. Representative images of three independent experiments are shown. The values represent the mean ± SD of results from three independent experiments. * *p* < 0.05 as compared the control group; # *p* < 0.05 as compared the treated group.

ROS play an important role in many physiological conditions along with pathological conditions, such as endometriosis [33,34]. Thus, we examined the effect of iron overload on the intracellular levels of ROS using a fluorescence-sensitive probe, DCF-DA. The ROS levels were significantly increased by FeSO_4_ and FAC treatment in 12Z cells (Figure 4). In addition, the antioxidant N-acetyl-L-cysteine (NAC) significantly suppressed the iron-stimulated migration of endometriotic 12Z cells (Figure 5A). Moreover, the iron-induced MMP-2/-9 expression was significantly suppressed by NAC treatment in 12Z cells (Figure 5B). These results suggest that iron-induced ROS may promote endometriosis progression.

### 3.5. Involvement of the NFκB Pathway in Iron-Induced Migration

**Figure 6:**
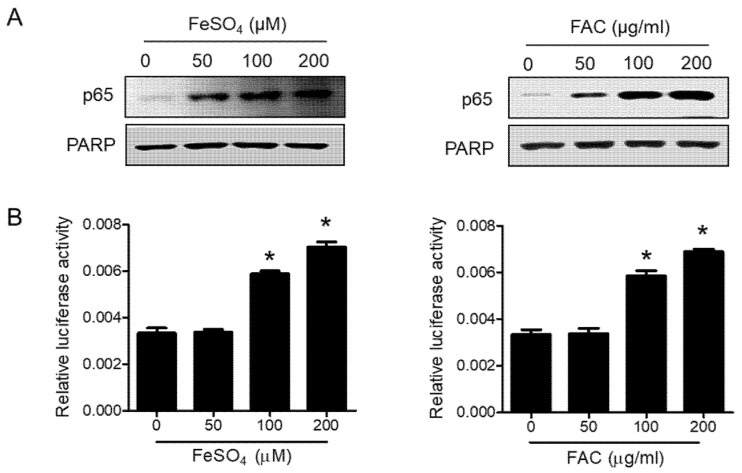
Effect of iron on the NFĸB pathway in human endometriotic cells. (**A**) Human endometriotic 12Z cells were treated with iron for 24 h. The nuclear expression of p65 was determined by Western blot analysis. PARP was used as a nuclear protein loading control. (**B**) 12Z cells were transiently transfected with pNFκB-Luc vector and then treated with iron for 6 h. Luciferase assay was performed. Representative images of three independent experiments are shown. Values represent the means ± SDs of three independent experiments. * *p* < 0.05 as compared with control.

**Figure 7:**
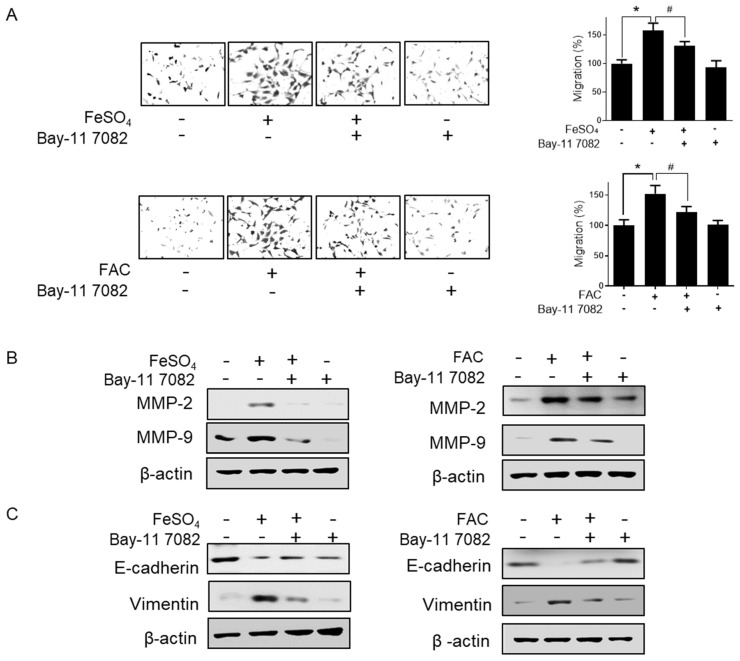
Effect of NFĸB inhibitor on iron-induced migration in human endometriotic cells. Human endometriotic 12Z cells were treated with iron (FeSO_4_: 100 µM; FAC: 100 µg/mL) in the presence of Bay 11-7082 (1 µM) for 24 h. (**A**) Transwell migration assay was performed. (**B**) MMP-2 and MMP-9 expressions were determined by Western blot analysis. (**C**) E-cadherin and vimentin expressions were determined by Western blot analysis. β-Actin was used as a loading control. Representative images of three independent experiments are shown. The values represent the mean ± SD of the results from three independent experiments. * *p* < 0.05 as compared to the control group; # *p* < 0.05 as compared to the treated group.

The NFκB pathway promotes cell invasion and migration in various cancers [35,36]. Thus, we determined the effect of iron overload on the NFκB pathway in 12Z cells. FeSO_4_ and FAC treatment significantly increased the nuclear localization of p65 (Figure 6A). In addition, a luciferase assay revealed that FeSO_4_ and FAC induced NFκB transcriptional activity in 12Z cells (Figure 6B). Next, we confirmed whether the activation of the NFκB pathway is involved in iron-induced migration in 12Z cells. Bay-11-7082, an NFκB inhibitor, significantly suppressed the iron-stimulated migration of endometriotic 12Z cells (Figure 7A). Furthermore, the pretreatment of 12Z cells with Bay 11-7082 significantly inhibited iron-induced MMP-2/-9 expression (Figure 7B). Furthermore, Bay 11-7082 reversed iron-suppressed E-cadherin expression and iron-stimulated vimentin expression (Figure 7C). These results suggest that iron promotes the migration of human endometriotic cells through activation of the NFκB pathway.

### 3.6. Effect of Iron Treatment on EMT and MMP-2/-9 Expression in Endometriotic Lesions in Mouse Model

**Figure 8:**
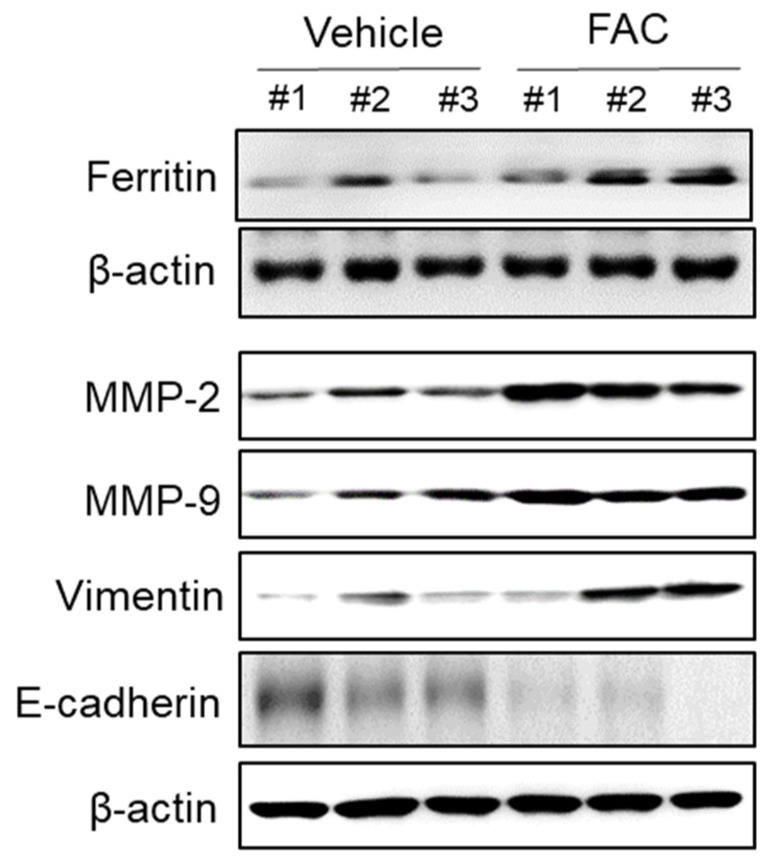
Effect of iron on EMT and MMP-2/-9 expression in endometriotic lesions of mouse model. Female BALB/c mice were intraperitoneally administered with vehicle (0.05% CMC) and FAC (75 mg/kg) three times per week for 5 weeks. The endometriotic lesion tissue on peritoneum was lysed and the protein expressions of ferritin, MMP-2, MMP-9, vimentin, and E-cadherin were determined by Western blot analysis. β-Actin was used as a loading control. Each mouse tissue of the vehicle and FAC groups was marked with a number (#1–3).

We investigated the effect of iron on endometriosis in an in vivo model. Iron did not induce a significant change in the total number and weight of endometriotic lesions after five weeks of treatment (Supplementary Figure S2). However, Western blot analysis revealed that iron treatment increased MMP-2/-9 and vimentin expression and decreased E-cadherin in the endometriotic lesions compared to vehicle control (Figure 8). These data suggest that iron may increase the migratory ability of endometriotic cells by the induction of EMT and the upregulation of MMPs in vivo.

## 4. Discussion

Cell migration can be increased by the excessive expression of MMPs [37], leading to the local destruction of the extracellular matrix and the establishment of early lesions [38]. In fact, the levels of MMP-2 and MMP-9 were found to be higher in the peritoneal fluid of women with endometriosis compared to that of healthy patients [39,40]. Here, we have demonstrated that iron promotes the migration of human endometriotic cells and MMP-2 and MMP-9 expressions in human endometriotic cells. These results suggest that enhanced MMP levels in endometriotic tissue may be associated with iron overload. Our data are consistent with reported findings showing the effect of iron on MMP expression in several different cell types. For example, iron enhanced the expression levels of MMP-9, resulting in the development and progression of head and neck squamous cell carcinoma, as well as the activation of microglial cells [41,42]. In addition, increased cellular iron levels were related to an increase in the expression of MMPs, including MMP-9, in activated microglial cells. Iron induces the overproduction of ROS via Fenton and Haber–Weis reactions [43]. Although many studies have suggested that iron overload may play a key role in endometriosis via excess redox stress, there is limited experimental evidence showing how iron-induced ROS are involved in the invasive and migratory characteristics of endometriotic cells. Here, we demonstrated that the ROS scavenger NAC significantly inhibited iron-stimulated migration, EMT and MMP upregulation in endometriotic cells. Our results suggest that oxidative stress induced by iron overload or an imbalance between reactive oxygen species and antioxidants may be implicated in endometriotic cell migration. Our next question was how ROS can regulate migration-related gene expression in endometriotic cells.

NFĸB has been implicated in the regulation of many genes that are critical to the initiation and establishment of the early and late stages of endometriosis [44]. For example, inflammation mediators such as CCL2 have been found to be partially regulated by NFκB in endometriosis [45,46]. Furthermore, NFκB is known to act as a transcription factor for MMPs, which play a key role in cell invasion and migration [42]. In fact, the constitutive activation of NFκB has also been demonstrated in endometriotic lesions and peritoneal macrophages of endometriosis patients. In addition, agents blocking NFκB are effective inhibitors of endometriosis development, and some drugs that inhibit NFκB have proven to be efficient at reducing endometriosis-associated symptoms in women [44]. Interestingly, iron overload has been suggested to trigger and maintain the NFκB constitutive activation shown in peritoneal endometriotic lesions, as well as activate NFκB in refluxed endometrial cells during menstruation, increasing the inflammatory response by ectopic endometrial cells [5,47,48]. In this regard, we investigated the involvement of the NFκB pathway in the iron-induced migration of endometriotic cells. Bay-11-7082, a NFκB inhibitor, significantly suppressed iron-stimulated migration and migration-related gene expression in endometriotic 12Z cells.

Based on our findings, both ROS upregulation and NFκB activation seem to be required for iron-induced migration and the related gene expression, including of MMPs. Notably, elevated ROS levels are responsible for the constant activation of transcription factors, including NFκB [49]. ROS have been demonstrated to trigger the activation of the NFκB pathway by increasing the phosphorylation of IκB, and are implicated in the development of several pathological conditions [50]. These data suggest that ROS upregulation is required for NFκB activation by iron in endometriotic cells. Thus, we confirmed whether ROS acts upstream of NFκB using the ROS scavenger NAC. NAC markedly inhibited the iron-stimulated phosphorylation of p65, an indicator of NFκB activation, in endometriotic cells (Supplementary Figure S3).

It has been demonstrated that the epithelial cells of endometriotic primary cells are invasive and play a key role in the development and invasion of endometriosis [51,52]. In addition, EMT, where immotile epithelial cells attain phenotypes of motile mesenchymal cells, has been implicated in the development and progression of endometriosis [18,53,54]. In this regard, we have investigated the effect of iron on human endometriotic epithelial 12Z cells in this study, and demonstrated that iron treatment significantly increased the migration abilities of human endometriotic epithelial cells. In the animal study, we observed increased EMT and MMP-2/-9 expressions not in the epithelial portion of endometriotic lesions, but in whole lesions. Considering stromal cells are the major type of cells of endometriotic lesions, the effect of iron on endometriotic stromal cells should be further investigated.

Here, we investigated the expression of the iron storage protein, ferritin, in endometriotic cells to establish the possible role of iron in endometriosis. Ferritin was demonstrated to be overexpressed in endometriotic tissues in patients with endometriosis. Interestingly, in some cancer cells, ferritin expression was demonstrated to be regulated by inflammatory cytokines through oxidative stress and NFκB activation. [55]. In this regard, further studies are needed to investigate whether iron-induced oxidative stress and NFκB activation are responsible for ferritin overexpression in endometriotic cells, or whether other molecular mechanisms are associated with iron induced-ferritin expression in endometriosis.

## Supplementary Materials

The following are available online at https://www.mdpi.com/2227-9059/8/11/454/s1, Figure S1: Effect of FeSO_4_ and FAC on viability of 12Z cells, Figure S2: Effect of FAC on endometriotic lesion formation in female BALB/c mice, Figure S3: Effect of NAC on iron-induced activation of NFκB in 12Z cells.
