# Supplementary material for: Iron-Storage Protein Ferritin Is Upregulated in Endometriosis and Iron Overload Contributes to a Migratory Phenotype
Source: Biomedicines. 2020 Oct 27;8(11):454. doi: 10.3390/biomedicines8110454 (PMC7694081; doi:10.3390/biomedicines8110454)
Supplement: Supplementary file 1 [file biomedicines-08-00454-s001.pdf]

biomedicines

MDPI

Article

Iron-Storage Protein Ferritin is Upregulated in Endometriosis and Iron Overload Contributes to a Migratory Phenotype

Jeong-Hwa Woo , Youn Seok Choi and Jung-Hye Choi

Methods

MTT Assay

To analysis cell viability, 3-(4,5-dimethylthiazol-2-yl)-2,5-diphenyl-tetrazolium bromide (MTT) assay was carried out. Cells were plated in a 96-well plate (5×10^4 cells/well) and incubated overnight to allow adhesion of the cells. After treatment with $FeSO_4$ and FAC, the cells were incubated for 24 h. Then, 50 μL of MTT solution (1 mg/mL stock solution) was added into each well and the plate was incubated for another 3 h. Formazan crystal formed in the cells was dissolved in DMSO after removal of culture medium. The absorbance at 540 nm was measured in a microplate reader (SpectraMax; Molecular Devices, Sunnyvale, CA, USA).

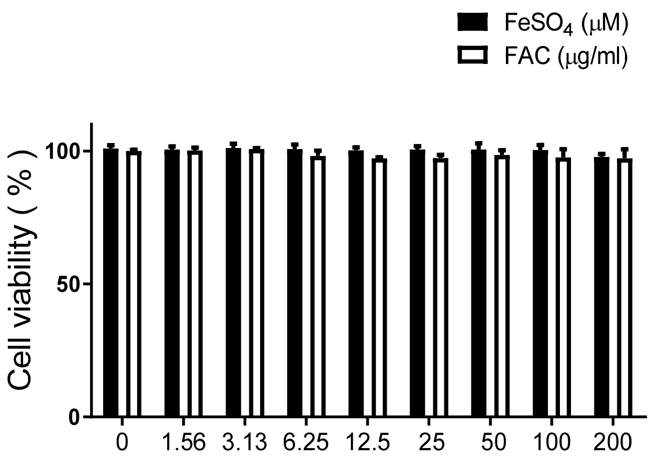

Figure S1. Effect of $FeSO_4$ and FAC on viability of 12Z cells. 12Z cells were treated with various concentration of $FeSO_4$ and FAC for 24. MTT assay were performed.

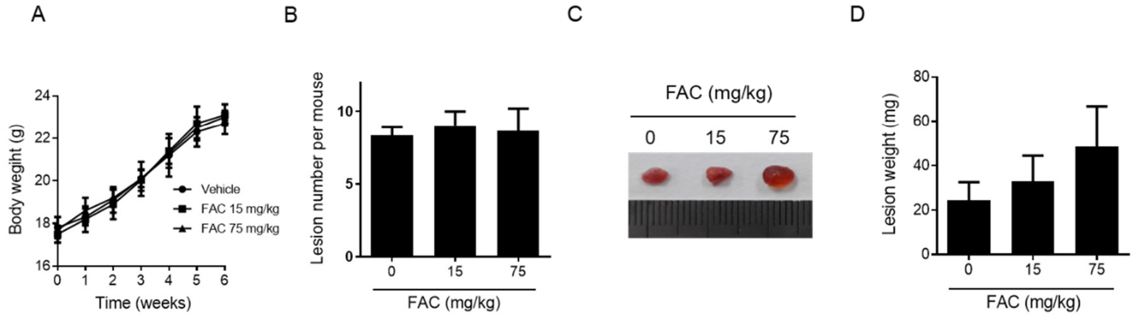

Figure S2. Effect of FAC on endometriotic lesion formation in female BALB/c mice. Female BALB/c mice were intraperitoneally administered with vehicle (0.05% CMC), FAC (15mg/kg) or FAC (75 mg/kg) three times per week for 5 weeks. (**A**) Body changes were measured every week. (**B**) Numbers of endometriotic lesions on the peritoneum and visceral organs. (**C**) Representative image of endometriotic lesions (**D**) Weight of endometriotic lesions.

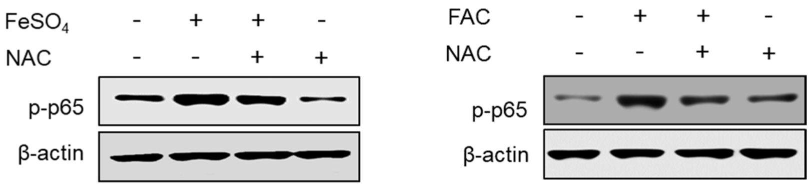

Figure S3. Effect of NAC on iron-induced activation of NFκB in 12Z cells. Human endometriotic 12Z cells were treated with iron ($FeSO_4$: 100 μM, FAC: 100 μg/ml) in the presence of NAC for 24 h. p-p65 expression was determined by Western blot analysis. β-Actin was used as loading control. Representative images of three independent experiments are shown.

© 2020 by the authors. Licensee MDPI, Basel, Switzerland. This article is an open access article distributed under the terms and conditions of the Creative Commons Attribution (CC BY) license (http://creativecommons.org/licenses/by/4.0/).
